# Ecological Contexts of Index Cases and Spillover Events of Different Ebolaviruses

**DOI:** 10.1371/journal.ppat.1005780

**Published:** 2016-08-05

**Authors:** Seth D. Judson, Robert Fischer, Andrew Judson, Vincent J. Munster

**Affiliations:** 1 Virus Ecology Unit, Laboratory of Virology, Division of Intramural Research, National Institute of Allergy and Infectious Diseases, National Institutes of Health, Rocky Mountain Laboratories, Hamilton, Montana, United States of America; 2 David Geffen School of Medicine at UCLA, Los Angeles, California, United States of America; 3 Square Inc, San Francisco, California, United States of America; Division of Clinical Research, UNITED STATES

## Abstract

Ebola virus disease afflicts both human and animal populations and is caused by four ebolaviruses. These different ebolaviruses may have distinct reservoir hosts and ecological contexts that determine how, where, and when different ebolavirus spillover events occur. Understanding these virus-specific relationships is important for preventing transmission of ebolaviruses from wildlife to humans. We examine the ecological contexts surrounding 34 human index case infections of ebolaviruses from 1976–2014. Determining possible sources of spillover from wildlife, characterizing the environment of each event, and creating ecological niche models to estimate habitats suitable for spillover, we find that index case infections of two ebolaviruses, Ebola virus and Sudan virus, have occurred under different ecological contexts. The index cases of Ebola virus infection are more associated with tropical evergreen broadleaf forests and consuming bushmeat than the cases of Sudan virus. Given these differences, we emphasize caution when generalizing across different ebolaviruses and that location and virus-specific ecological knowledge will be essential to unravelling how human and animal behavior lead to the emergence of Ebola virus disease.

## Author Summary

Multiple Ebola virus disease outbreaks have occurred over the past 40 years, yet we still do not know the geographical distributions, definitive host species, and suitable habitats for animal-to-human transmission of different ebolaviruses. Each Ebola virus disease outbreak has started with at least one transmission event from a wildlife host to a human, also known as a spillover event. While researchers have studied these events in regards to Ebola virus disease outbreaks, many studies neglect that there are multiple ebolaviruses and that these viruses may differ in their spillover events. We characterize the specific ecological contexts of different ebolavirus spillover events based on recorded index case infections. Comparing the environmental contexts of these cases and using ecological niche modelling, we find that two ebolaviruses have different suitable habitats for spillover. The different habitats and contexts of the two ebolaviruses involved in the majority of outbreaks, Ebola virus and Sudan virus, indicate that we must further investigate virus-specific differences in ebolaviruses and their hosts.

## Introduction

From the first recognized outbreak of Ebola virus disease (EVD) in 1976 to the recent outbreak beginning in 2013, our knowledge about the molecular biology and epidemiology of viruses belonging to the genus *Ebolavirus* has increased dramatically. Yet after nearly 40 years of research, we still have a limited understanding of the ecology and evolution of these viruses outside the context of outbreaks in humans [1]. One limitation in understanding ebolavirus ecology has been identifying the reservoir hosts in which ebolaviruses persist in nature, while another obstacle has been determining what causes the sporadic transmission of ebolaviruses from their natural reservoir into other animals and humans, leading to subsequent human-to-human transmission and outbreaks of EVD. These initial episodes of animal-to-human transmission are called spillover events, and knowing when, where, and under what environmental conditions ebolavirus spillovers occur could reveal underlying relationships in ebolavirus ecology. Additionally, identifying host-pathogen interactions of ebolaviruses with their natural reservoir and spillover hosts, as well as the interactions of these hosts with humans, could help researchers improve preemptive measures for transmission from wildlife as well as answer fundamental questions about virus ecology.

The genus *Ebolavirus* belongs to the family *Filoviridae* along with the genera *Cuevavirus* and *Marburgvirus*. Five species of viruses have been established in the genus *Ebolavirus*: *Zaire ebolavirus*, *Bundibugyo ebolavirus*, *Sudan ebolavirus*, *Taï Forest ebolavirus*, and *Reston ebolavirus*. The viruses belonging to these species are known as Ebola virus (EBOV), Bundibugyo virus (BDBV), Sudan virus (SUDV), Taï Forest virus (TAFV), and Reston virus (RESTV), respectively [2]. These different ebolaviruses are genetically distinct, with SUDV and RESTV being the most divergent from the other ebolaviruses [3]. Factors influencing the speciation of ebolaviruses and how ebolavirus speciation relates to reservoir host evolution and ecology remain enigmatic.

No ebolavirus has ever been isolated from a putative reservoir species. In addition, of all the ebolaviruses, only EBOV has had its RNA detected in potential reservoir hosts, 3 species of African fruit bats [4]. Duikers (*Cephalophus* species), gorillas (*Gorilla gorilla*), chimpanzees (*Pan troglodytes*), and various rodents have also tested positive for EBOV RNA [5,6]. It is widely suspected that these are probably incidental hosts that are indirectly infected by bats [7]. While serological evidence exists for RESTV in Asian bats and TAFV was found in a deceased chimpanzee, both SUDV and BDBV have yet to be identified via serology or PCR in any wildlife [7]. Therefore, we do not know the definitive reservoir host species for any ebolavirus or what factors influence ebolavirus transmission from wildlife into human populations.

While the difficulty of detecting ebolaviruses in wildlife reservoirs hinders the identification of reservoir hosts and the determination of their enzootic cycles, examining the ecology of ebolaviruses at the human-animal interface could yield insights about potential animal hosts as well as the ecological conditions that drive the emergence of these pathogens. EBOV, SUDV, BDBV, and TAFV are known to cause EVD in humans. Antibodies to RESTV have been detected in humans in the Philippines [8]; however no RESTV spillover events in humans have been documented, and it is assumed that RESTV is nonpathogenic in humans [9]. Therefore, outbreaks of EVD in human populations have enabled researchers to characterize the other four ebolaviruses according to their locality, case fatality, and epidemiology [10], as well as understand human-to-human transmission [11]. However, the individual spillover events that lead to outbreaks in humans have not been as well characterized. While few ebolavirus spillover events have been confirmed, there are reported index cases, the first human cases to be clinically described or laboratory confirmed in a chain of transmission. The central estimate for the incubation period until onset of EVD is 5.3–12.7 days for EBOV, 3.35–12 days for SUDV, and 6.3–7 days for BDBV [12]. Therefore, these index cases provide an approximation of roughly where and when spillover events have occurred. Since only EBOV has been sparsely detected in wildlife, examining these spillover events via index case reports is currently the only way that we can consistently compare the ecologies of multiple ebolaviruses.

In order to specify the ecological contexts of ebolavirus spillover events, one must first define the habitats where spillover events occur. Ecological niche models (ENMs) can be used to qualitatively compare the habitats where different species occur and identify regions of habitat suitability [13]. This toolset is increasingly being used to predict the ecological niches of viruses. For example, cases of human monkeypox disease have been used to model the ecological niches of monkeypox virus [14, 15]. Instead of using species occurrences and predicting fundamental ecological niches, we can use the locations of ebolavirus index cases and their associated spillover events from wildlife into humans to determine suitable habitats for ebolavirus spillover. Comparing the suitable spillover habitats of different ebolaviruses allows us to further compare the ecological contexts of multiple ebolaviruses and determine virus-specific factors of spillover.

Here we characterize the habitat and context of all known ebolavirus index case infections and associated spillover events into humans from 1976–2014 to investigate species and location specific ecological relationships. We use an ENM modeling approach that is optimized for small sample sizes to compare the habitats of spillover events of different ebolaviruses. In doing so, we find that distinct ebolaviruses spill over into humans under specific ecological contexts and are associated with different habitats.

## Results

### Ebolavirus Index Cases and Spillover Events

**Fig 1:**
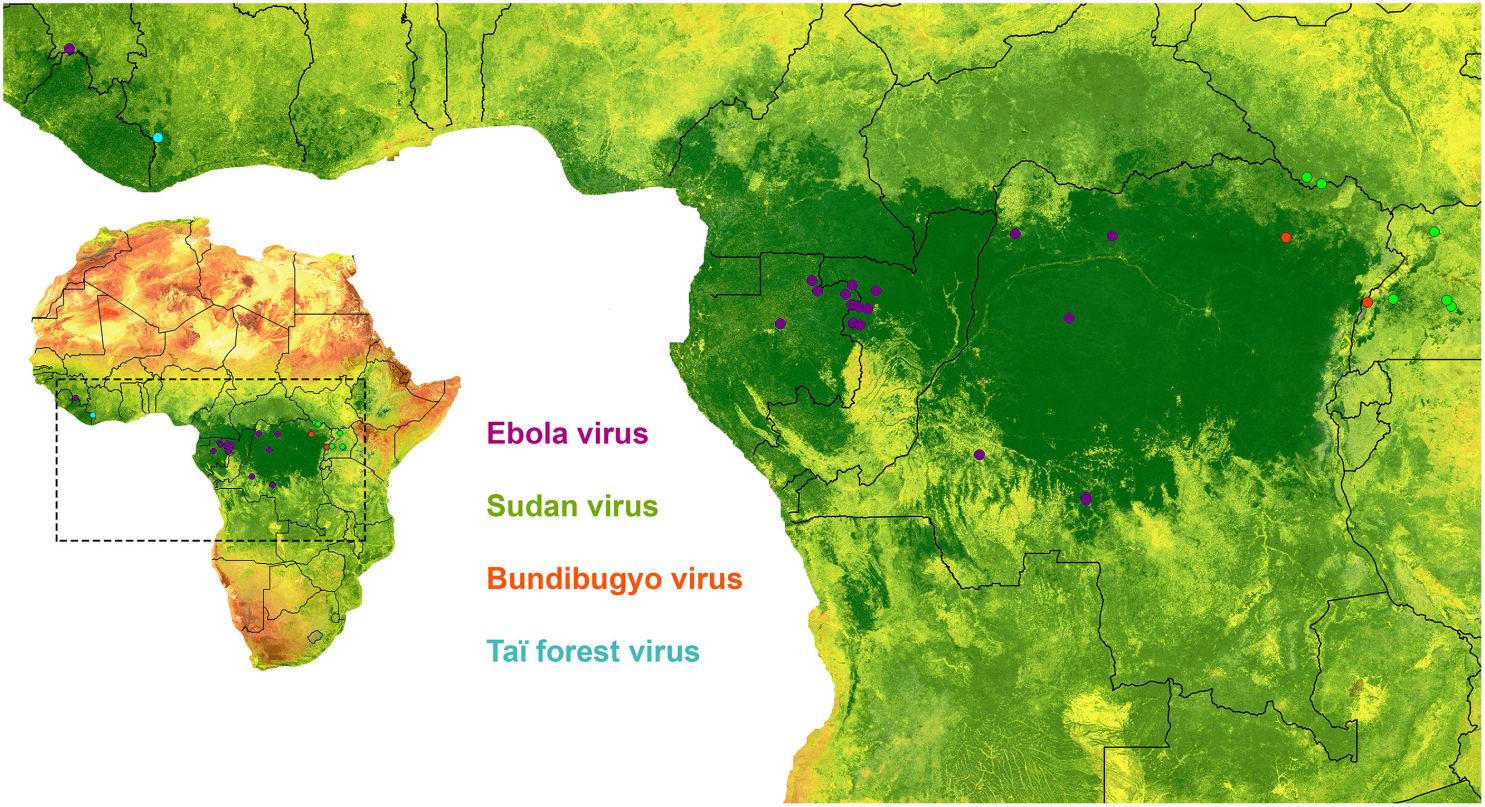
Locations of ebolavirus index cases 1976–2014.

We identified a total of 34 index cases and the associated spillover events of four ebolaviruses (24 EBOV, 7 SUDV, 2 BDBV, and 1 TAFV) (Table 1). We hereafter refer to both these index cases and their associated spillover events as “spillover events.” Spillover events of viruses from each species occurred in distinct geographic locations (Fig 1), while 1 SUDV and 4 EBOV spillover events occurred in the same location as a previous event.

**Table 1:** Ebolavirus index cases and associated spillover events 1976–2014.

Country	Location	Index case date*	Index patient	Potential source of spillover	Season^†^	Ebolavirus	Lat	Long	Reference
South Sudan	Nzara	6/27/1976	male, textile worker	insectivorous bats (*M*. *condylurus*), rodents (*Rattus rattus*)	Wet	SUDV	4.63912	28.25115	[30, 38]
DRC	Yambuku	9/1/1976	44 y/o male, teacher	antelope, monkey meat	Wet	EBOV	2.82535	22.22567	[52]
DRC	Bonduni village	June/1977	9 y/o female		Wet	EBOV	2.88874	19.22384	[53]
South Sudan	Nzara	7/31/1979	male, textile worker	insectivorous bats (*M*. *condylurus*), rodents (*Rattus rattus*)	Wet	SUDV	4.63912	28.25115	[54]
Gabon	Mekouka, Andock mining camps	11/13/1994	gold miner		Wet	EBOV	1.44201	12.92929	[55]
Cote d'Ivoire	Tai National Park	11/16/1994	34 y/o female ethologist	chimpanzee (*Pan troglodytes*) carcass	Dry	TAFV	5.86442	-7.31794	[39, 56]
DRC	Mwembe, Kitwit	1/6/1995	42 y/o male farmer, charcoal pit worker		Wet^‡^	EBOV	-3.951	18.115	[57]
Gabon	Mayibout 2	1/31/1996	butcher	chimpanzee carcass	Lesser dry	EBOV	1.11667	13.1	[39, 55]
Gabon	Logging camp near Boue	7/13/1996	hunter	chimpanzee carcass	Dry	EBOV	0.1	11.95	[39, 55]
Gabon	Logging camp near Boue	8/24/1996	hunter	chimpanzee carcass	Dry	EBOV	0.1	11.95	[39, 55]
Uganda	Rwot-Obilo village, Gulu	8/30/2000			Wet	SUDV	2.94998	32.19997	[41, 58]
Gabon	Mendemba	Oct/2001		duiker (*Cephalophus* sp.) or gorilla (*Gorilla gorilla*) carcass	Wet	EBOV	0.70055	14.15543	[39, 41]
Gabon	Mendemba	10/25/2001		duiker or gorilla carcass	Wet	EBOV	0.70055	14.15543	[40, 41]
Gabon	Ekata	11/28/2001		duiker carcass	Wet	EBOV	0.67705	14.28902	[40, 41]
Gabon & RoC	Olloba	12/1/2001		gorilla carcass	Lesser dry	EBOV	0.62049	14.37774	[40, 41]
Gabon	Ekata	12/22/2001			Lesser dry	EBOV	0.67705	14.28902	[40, 41]
Gabon	Etakangaye	12/29/2001		chimpanzee carcass	Lesser dry	EBOV	1.0166	13.966	[40, 41]
RoC	Entsiami	Jan/2002			Dry	EBOV	0.09141	14.21818	[40, 41]
Gabon & RoC	Olloba	5/17/2002		chimpanzee carcass, pangolin	Wet	EBOV	0.62049	14.37774	[40, 59]
Gabon	Grand Etoumbi	4/27/2002	hunter	gorilla carcass	Wet	EBOV	1.30411	14.17743	[39]
RoC	Yembelangoye village	12/21/2002		gorilla carcass	Lesser dry	EBOV	0.13418	14.20981	[39, 60]
RoC	Mvoula	1/1/2003		chimpanzee carcass	Wet^‡^	EBOV	0.06823	14.41997	[39, 60]
RoC	Mbandza village	10/11/2003		monkey carcass (*Cercopithecus nictitans*)	Wet	EBOV	0.56015	14.65732	[39, 61]
South Sudan	Forests bordering Yambio	4/15/2004	hunter	baboon carcass (*Papio* sp.)	Wet	SUDV	4.43149	28.7054	[39]
RoC	Parc d'Odzala	4/18/2005	hunter	duiker or gorilla carcass	Wet	EBOV	1.12508	14.9158	[39, 60]
DRC	Bamoukamba 2	5/15/2007	butcher	fruit bat carcass (*H*. *monstrosus*, *E*. *franqueti*)	Dry	EBOV	-5.25956	21.40954	[39, 62]
Uganda	Kabango village	8/20/2007	26 y/o female		Wet	BDBV	0.7706	30.13041	[39, 63]
DRC	Luebo	11/27/2008	18 y/o pregnant female		Wet	EBOV	-5.35063	21.41646	[62, 64]
Uganda	Nakisamata village	5/1/2011	12 y/o female		Wet	SUDV	0.641297	32.71896	[65]
DRC	Isiro	June/2012			Wet	BDBV	2.772236	27.60828	[38, 66]
Uganda	Nyanswiga (Kibaale)	6/11/2012			Dry	SUDV	0.86599	30.92654	[39, 66]
Uganda	Luwero district	11/13/2012			Wet	SUDV	0.83175	32.58253	[39, 66]
Guinea	Meliandou	12/2/2013	2 y/o male	insectivorous bats (*M*. *condylurus*)	Dry	EBOV	8.616067	-10.0612	[38, 67]
DRC	Boende	7/26/2014	pregnant female butcher	monkey carcass	Wet	EBOV	0.284286	20.88509	[38, 68]

*Month shown for index cases without exact date

^†^ Dry season = monthly precipitation < 60 mm, Lesser dry = 60 mm < monthly precip. <120 mm, Wet = monthly precip. >120 mm

^‡^Actual month was atypically wet or dry compared to long-term monthly mean

EBOV spillover events have occurred at latitudes ranging from -5.3°-8.6° throughout the year during both wet and dry seasons. SUDV spillover events were more spatially clustered at .64°-4.6° and 6/7 (86%) occurred during the wet season. Two SUDV spillovers occurred in the same location during the same season within 3 years from each other. The two BDBV events occurred at .77°-2.7° during the wet season, and the TAFV event also occurred during the wet season.

### Suitable Habitats for Ebolavirus Spillover

**Fig 2:**
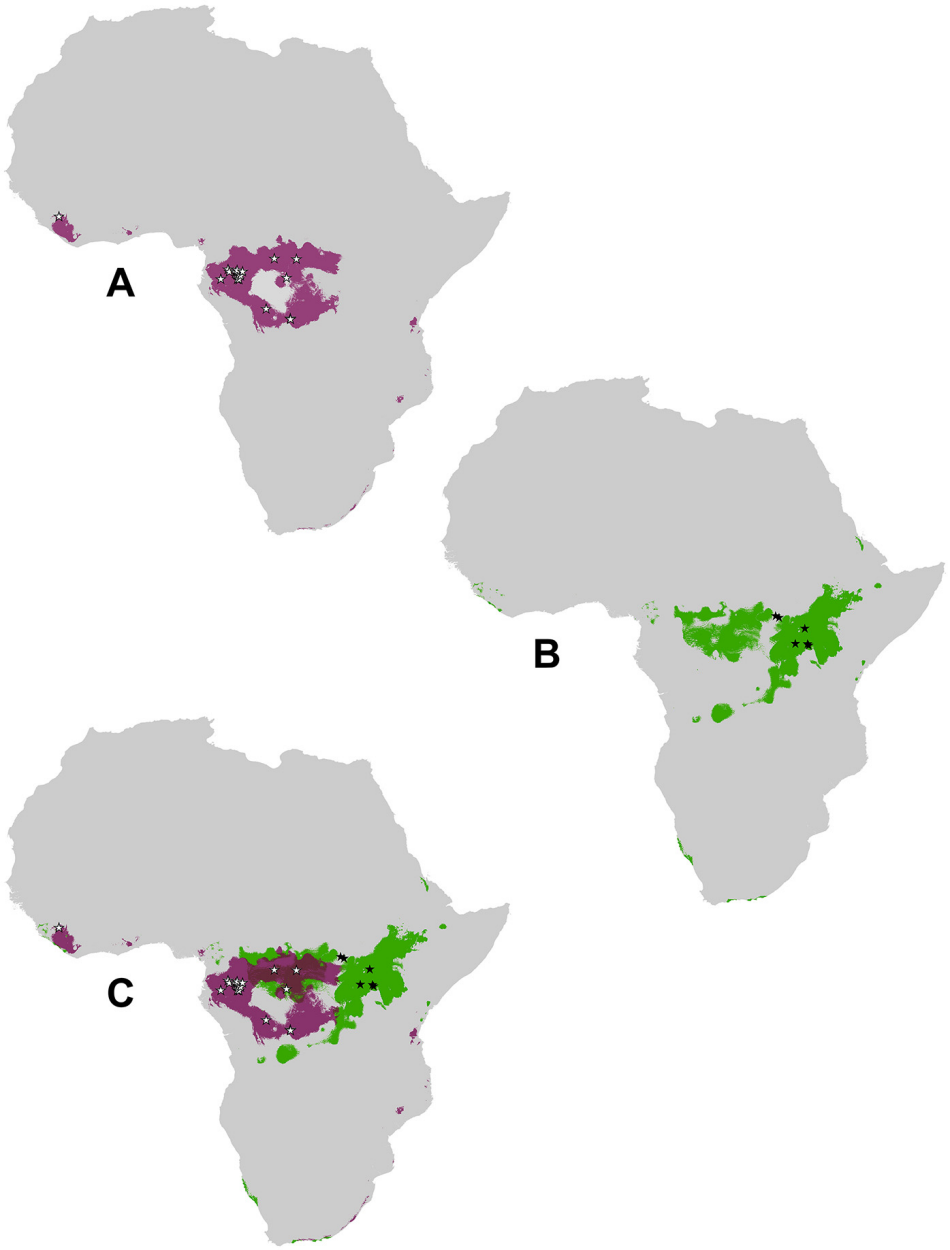
Suitable habitats for EBOV and SUDV spillovers events in mainland Africa. MaxEnt models with a minimum training presence threshold were used to determine the habitat suitability for EBOV and SUDV based on spillover locations. A) Purple represents EBOV model, and white stars indicate EBOV spillover locations. B) Green represents SUDV model, and black stars indicate SUDV spillover locations. C) Overlap of EBOV and SUDV models. See Fig A in S1 Text for global habitat suitability.

We used the locations of ebolavirus spillover events and environmental covariates to create ecological niche models, which identified habitats similar to those where different ebolaviruses have spilled over into humans. Suitable habitats for EBOV and SUDV spillover events within Africa are shown in Fig 2. These models were made under the assumption that EBOV and SUDV spillovers from wildlife do not occur outside of mainland Africa. Additional models were made to compare the habitats of EBOV and SUDV spillover events within a global context (Fig A in S1 Text). Due to the limited sample size of TAFV and BDBV spillover events, we could not create models for these species that were statistically significant. The minimum training presence threshold was chosen to create the binary maps because it was more liberal than the 10 percentile training presence threshold. The minimum training presence threshold is established by the lowest habitat suitability in the training data set; therefore, all indicated regions have ecological conditions that at minimum match those in the least suitable confirmed location of spillover.

The models for EBOV and SUDV at the minimum training presence threshold successfully predicted spillover event locations at high rates, 85% (17/20) and 66% (4/6) respectively. A *P* value of 3e-05 was calculated for the SUDV models and 3e-19 for the EBOV models, indicating that both models were statistically significant at predicting distribution of spillover events compared to random.

The models of EBOV and SUDV spillovers at the minimum training presence threshold overlapped in approximately 12% of their total area. No SUDV spillover events were within the model of EBOV, and only three EBOV events occurred within the model of SUDV. Of the original 20 environmental covariates, 9 were determined to be important in contributing to the models of both ebolaviruses (Table 2).

**Table 2:** Environmental covariates used to create ENMs.

Environmental Covariate	Important for SUDV or EBOV ENM
Annual Mean Temperature	
Mean Diurnal Range	Both
Isothermality	Both
Temperature Seasonality	Both
Max Temperature of Warmest Month	
Min Temperature of Coldest Month	Both
Temperature Annual Range	EBOV
Mean Temperature of Wettest Quarter	
Mean Temperature of Driest Quarter	
Mean Temperature of Warmest Quarter	
Mean Temperature of Coldest Quarter	
Annual Precipitation	
Precipitation of Wettest Month	Both
Precipitation of Driest Month	
Precipitation Seasonality	Both
Precipitation of Wettest Quarter	
Precipitation of Driest Quarter	
Precipitation of Warmest Quarter	EBOV
Precipitation of Coldest Quarter	
Elevation	Both

### Terrain, Vegetation, and Climate

SUDV spillover events occurred at a significantly higher mean elevation than those of EBOV (p = 0.004). The 95% CI for the difference in means of SUDV and EBOV events was 196–671 m. EBOV events are also more associated with evergreen broadleaf forest compared to other land cover types than SUDV events (p = 0.0007), whereas SUDV events are more associated with woodland (p = 0.0078).

**Fig 3:**
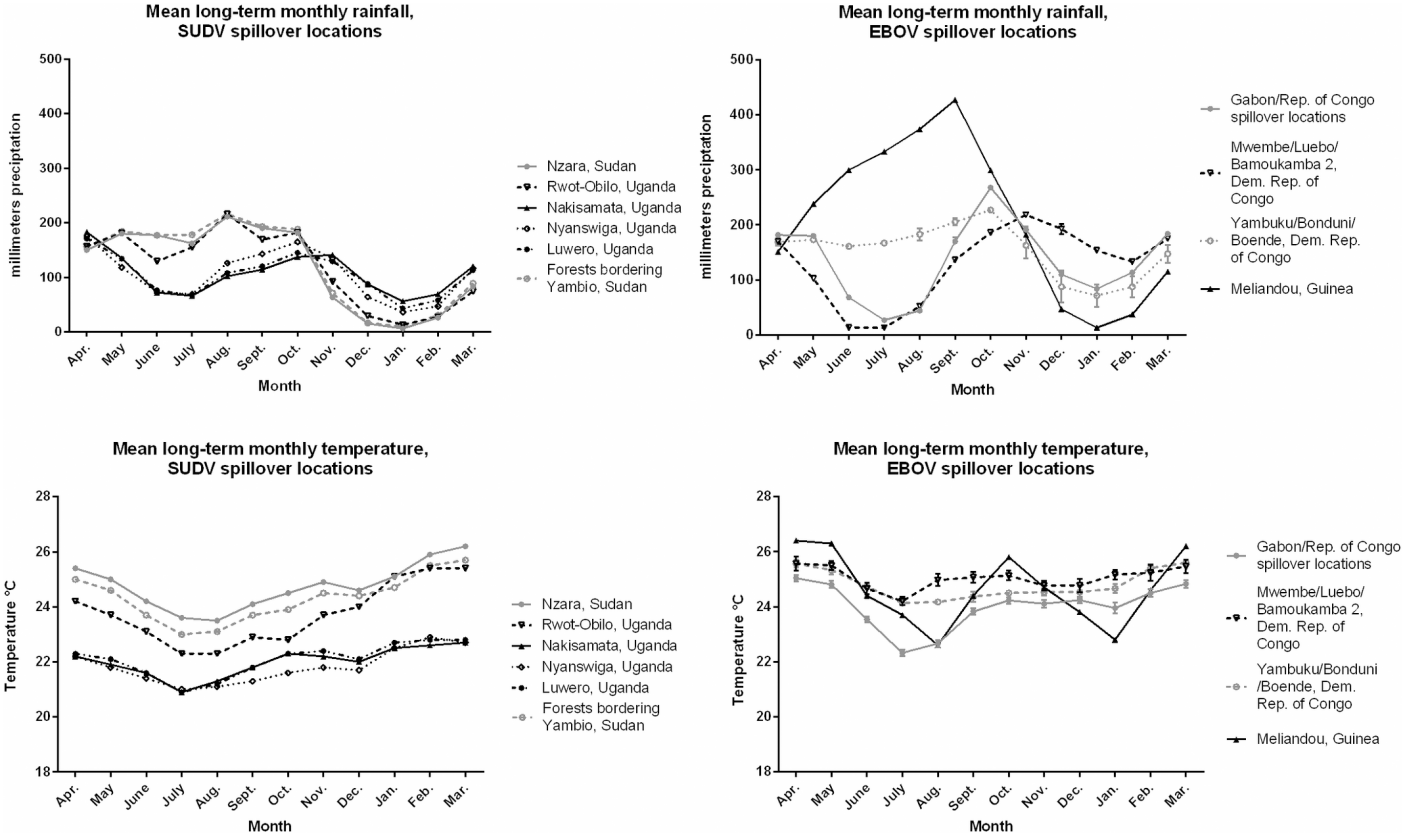
Monthly rainfall and temperature in SUDV and EBOV index case locations. The mean long-term monthly rainfall and temperature for the six SUDV spillover locations are depicted. For the twenty EBOV spillover locations, the mean rainfall and standard error of the mean are shown for locations that were in proximity to each other.

The long-term monthly mean rainfall and temperature varied between SUDV and EBOV locations (Fig 3). Comparing SUDV and EBOV spillover locations, there was no significant difference in the mean temperature (p = 0.18) or rainfall (p = 0.95) of the actual month when an event occurred.

### Human-Wildlife Interactions

The suspected animal sources of all known spillover events of viruses from different *Ebolavirus* species are shown in Table 1. The geographic distributions of these animals from the IUCN Red List [16] in relation to EBOV and SUDV ENMs are in Figs B-D in S1 Text. EBOV spillover events were more associated with bushmeat contact than SUDV spillover events (p = 0.012). Chimpanzees, gorillas, duikers, monkeys, and fruit bats were all suspected sources of spillover for EBOV. Only one SUDV spillover event could be potentially linked to the bushmeat of a baboon, a species not found to be associated with EBOV spillover. In the majority of SUDV spillover events, no possible animal source could be identified. Two SUDV spillover events were linked to the same factory containing insectivorous bats and rodents.

## Discussion

The recent outbreak of EVD has inspired research across many fields, so it is critical to communicate that multiple ebolaviruses can cause EVD outbreaks and could have distinct ecological relationships. Our findings demonstrate that the spillover events of different ebolaviruses do occur within specific ecological contexts and habitats. EBOV spillovers have occurred within or on the edges of tropical evergreen broadleaf forests, and index patients are often hunters, villagers, or outdoor workers who have come into contact with animals such as bats, primates, and duikers. In contrast, SUDV spillover events occur at higher elevations, are more associated with woodlands, and have cryptic animal sources of spillover. In order to study the ecological contexts of TAFV and BDBV, more information on their possible animal reservoirs and spillover events are necessary.

Our study provides an approximation of the different ecological contexts of the index cases and spillover events of four ebolaviruses known to cause EVD in humans. We compared the contexts of EBOV and SUDV spillover events as well as qualitatively estimated areas of habitat suitability for spillover of these viruses. We did not attempt to determine the fundamental ecological niches of different ebolaviruses and *Ebolavirus* species. Instead, we compared the ecology of ebolaviruses based on the contexts of index cases and associated spillover events. Our models indicate habitats that are similar to those where index cases have occurred. A biogeographical study with additional assumptions about ebolaviruses and their hosts could quantitatively compare whether the differences in EBOV and SUDV spillover event locations are due to differences in niche conservatism or differences in the distributions of spillover events.

One limitation to our approach is that spillover event locations are unlikely to be independent and index case reports may be subject to geographical and temporal variation in reporting bias. Therefore, our models could exclude actual habitats of these viruses. For instance, more EBOV spillover events have been detected in the western Congo Basin than in the central Congo Basin. Thus, parts of the central Congo Basin are excluded from our models despite that this region includes putative EBOV host ranges (Figs B-D in S1 Text). Our models may also include habitats that are ecologically suitable based on spillover events but are unlikely to contain the viruses. For example, we identified habitats in Southern Africa that have suitable ecological conditions but are geographically isolated from where ebolaviruses have so far been detected.

A further limitation to our study is that we used index case reports to approximate when spillover events occurred and analyzed the mean environmental data from the month of symptom onset or date of death, if symptom onset date was unavailable. Therefore, the variable duration of illness and incubation periods for ebolaviruses may influence our estimations of spillover event locations and results about seasonality. Additionally, historical monthly precipitation data was limited in spillover locations, so we used coarse resolution data to classify seasons and compare the precipitation within the month of a spillover event. Long-term monthly mean and bioclimatic data were available at much higher resolutions and were used for our other analyses. Until more ebolavirus spillover events are confirmed, our study provides an approximation of the ecological conditions of spillover events of ebolaviruses.

Despite these limitations, our study further supports that researchers cannot generalize about the ecological contexts of different ebolaviruses. Previous authors have used climatic data and ENMs to make inferences about spatial and temporal relationships of ebolaviruses. One group used NVDI models and Landsat data and found that the 1994–1996 EVD outbreaks occurred in tropical forest and were associated with climate changes from drier to wetter conditions [17]. In contrast, another group found that 1994–2002 EVD outbreaks were associated with drier conditions at the end of the rainy season [18], while another study found EVD outbreaks to be associated with lower temperatures and higher humidity [19]. These studies did not differentiate between different ebolaviruses, which may explain their discrepancies. Considering the different contexts of SUDV and EBOV spillover events, we found no associations between the temperature or precipitation during the month of a spillover event, and spillovers of both ebolaviruses occurred in both wet and dry seasons.

We also find that generalizing across ebolaviruses when making ENMs can miss virus-specific relationships. For instance, one group used the occurrence data of the 3 species of Old World fruit bats that were positive for EBOV RNA, EBOV infections in wildlife, and the locations of outbreaks associated with multiple ebolaviruses to create predictive risk maps for EVD [20]. This approach assumes that the reservoir species for different ebolaviruses are the same and that the spillover events of these viruses occur under the same ecological conditions. However, another group used the occurrence data from 12 EVD outbreaks in the period of 1976–2002 to create ENMs and found that eliminating SUDV occurrence data from the other ebolaviruses created a prediction that did not include the distribution of SUDV [21]. Using a modeling approach that was optimized for small sample sizes and spillover locations from 1976–2014, we corroborate this observation that EBOV and SUDV are associated with different habitats and may need to be considered separately in further ecological modeling.

The habitats suitable for SUDV and EBOV spillovers correspond with the serological evidence of these viruses in humans and wildlife. Our models showed that habitats in West Africa and Central Africa were suitable for EBOV spillover, while East Africa and parts of Central Africa were more suitable for SUDV spillover. Serological surveys in humans or animals have found antibodies to EBOV or SUDV in the majority of the countries identified by our models (Table 3). However, the cross-reactivity of different ebolavirus antibodies in these assays makes it difficult to distinguish the type of ebolavirus infection. As more seroepidemiological surveys are done and diagnostics improve, we can use this information to make more informed conclusions about where different ebolaviruses are found in humans and animals.

**Table 3:** African countries with suitable habitat for ebolavirus spillover and serological evidence.

Country	Type of ebolavirus habitat†	Serological evidence‡
Angola	SUDV	
Cameroon	EBOV, SUDV	EBOV, SUDV [69]
Central African Republic	EBOV, SUDV	EBOV, SUDV [69]
Democratic Republic of the Congo	EBOV, SUDV	EBOV [69]
Eritrea	SUDV	
Ethiopia	SUDV	EBOV [69]
Eritrea	SUDV	
Gabon	EBOV	EBOV [69]
Ghana	EBOV	EBOV [70]
Guinea	EBOV	EBOV [69]
Ivory Coast	EBOV	Unconfirmed ebolavirus type [69]
Kenya	SUDV	EBOV, SUDV [69]
Liberia	EBOV, SUDV	EBOV [69]
Mozambique	EBOV	
Namibia	SUDV	
Sierra Leone	EBOV, SUDV	EBOV [69]
South Africa	EBOV, SUDV	
South Sudan	SUDV	SUDV [69]
Tanzania	EBOV, SUDV	
Togo	EBOV	Unconfirmed ebolavirus type [69]

^†^Determined from ENM with minimum training presence threshold (Fig 2)

^‡^Due to possible assay cross-reactivity these may not be specific to type of ebolavirus

Understanding the ecological contexts of ebolavirus spillover events also allows us to infer about the potential geographic distributions of these viruses and their respective hosts. Our models support that there is ample suitable habitat for EBOV and SUDV spillover. The recent discovery of Lloviu virus, a related filovirus, in insectivorous *Miniopterus schreibersii* bats in Europe [22], the detection of filovirus RNA and antibodies in *Rousettus leschenaultii* in China and Bangladesh [23, 24], the circulation of RESTV in Southeast Asia [25, 26] and the recent emergence of EBOV in humans in West Africa suggest the possible circulation for filoviruses far beyond the areas with recorded EVD outbreaks. The lack of recorded spillover events in areas with suitable ecological conditions could therefore be due to the absence of pathogenic filoviruses and their respective hosts, lack of recognition of spillover events, absence of ecological and anthropogenic factors driving specific spillover events, or a combination of these factors.

Considering the different environments in which SUDV and EBOV spillovers have occurred, we can form two hypotheses about their distributions and reservoir hosts: SUDV and EBOV occupy different host species (potentially multiple species) with different habitats or SUDV and EBOV persist in the same species that is able to occupy multiple habitats. We can investigate these potential host relationships through comparing our models with the distributions of animal species that have been previously associated with ebolaviruses. The suitable habitats that we determined for EBOV and SUDV spillovers are shared among some potential bat hosts and are specific to others. The 3 species of fruit bats that were positive for EBOV RNA, *Hypsignathus monstrosus*, *Myonycteris torquata*, and *Epopmops franqueti*, have geographic ranges that overlap more closely with the tropical forests where EBOV spillovers have occurred, but the eastern boundaries of these species occur near SUDV spillover events as well [27] (Fig B in S1 Text). Additional African bat species have been identified as potential reservoirs for EBOV based on serology [7], but again the cross-reactivity of these assays makes it difficult to make associations with particular ebolaviruses. Of the distributions of serologically positive bat species (Fig C in S1 Text), those of *Micropteropus pusillus* and *Mops condylurus* best match the woodland habitat associated with SUDV [28, 29]. *M*. *condylurus* belongs to the family Molossidae, whereas the other potential ebolavirus hosts belong to Pteropodidae. Moreover, bats belonging to the same genus (*M*. *trevori*) were found in the textile factory in Nzara where at least two independent spillover events of SUDV occurred [30] and have a geographic distribution within the SUDV habitat (Fig D in S1 Text). Perhaps the evolutionary and ecological differences between molossid and pteropid bats could explain the divergence between SUDV and EBOV.

The hypothesis that different ebolaviruses may have different host species, and therefore different habitats suitable for spillover, is supported by *in vitro* and *in vivo* experiments. *In vitro* studies have shown that the receptor NPC1 influences filovirus susceptibility in different bat species [31]. These studies may be useful in determining whether particular bat species are capable reservoirs for different ebolaviruses. In addition, experimental infection studies showed efficient replication of Marburg virus, but limited replication of the five ebolaviruses in *Rousettus aegypticus* [32, 33], the reservoir host for Marburg virus. These findings highlight the potential for a single filovirus-single reservoir host species relationship, which may be why EBOV and SUDV spillovers occur in different habitats.

Different relationships of ebolaviruses with secondary hosts and regional human-animal interfaces could also explain the differing contexts of EBOV and SUDV events. The majority of the EBOV spillover cases came from infected primates, whereas the sources of SUDV were unidentified. Additionally, there have been no documented outbreaks of EVD in chimpanzees in East Africa near the habitat of SUDV, indicating that reservoir hosts of SUDV may not come into contact with wild apes. For example, western lowland gorillas and chimpanzees share *Ficus* spp. as a food source with the potential EBOV reservoir bat species *H*. *monstrosus* [34, 35], and such an epizootic link may not exist for SUDV and its reservoir host. Furthermore, other forest-dwelling animals, such as the bay duiker (*Cephalophus dorsalis*), are only associated with EBOV spillovers and have been positive for EBOV RNA, while the woodland savannah-inhabiting baboon (*Papio* sp.) has only been associated with an SUDV spillover. The different animal species that are associated with these two viruses and their spillovers further supports that these ebolaviruses may have different ecologies.

Lastly, our study also provides more evidence about the evolution of ebolaviruses. It has been previously noted that there is remarkably little genetic diversity between both spatially and temporally separated strains of the same ebolavirus [36]. We found relatively large and contiguous areas of suitable habitat for both EBOV and SUDV spillover, which might explain why genetically similar viruses can circulate across large distances. Meanwhile, isolates of EBOV and SUDV differ by more than 40% in their genomes on the nucleotide level [37], which could be explained by the small overlap in their spillover habitats and possible geographical isolation of their host species. Current phylogenetic trees place SUDV in a different clade than EBOV, and it is possible that geographic isolation led to this speciation, potentially due to the Albertine Rift [36], which is near the eastern border of the EBOV habitats in our models. More extensive sampling of ebolaviruses in wildlife and rapid identification of index cases will increase our understanding of ebolavirus ecology and evolution, as well as potentially guide preemptive control strategies.

Overall, we show that ecological contexts of ebolavirus spillover events are virus-specific, relating to particular habitats, animal distributions, and human activities. Therefore, researchers must be careful about generalizing about ebolaviruses and their ecologies. Uncovering nuances in virus ecology will require further explorations of the human-animal interfaces that lead to viral spillover and collaborations across disciplines.

## Methods

### Locating Spillover Index Cases

The geographic coordinates and identities of index cases were determined from the original literature describing EVD outbreaks and case reports (Table 1), using the Centers for Disease Control and Prevention’s EVD chronology as a guide [38], as well as a database of 22 EVD outbreaks [39]. Index cases were defined as patients who were the first to exhibit symptoms of EVD in an epidemic chain and had no previous contact with EVD patients. Additionally, index cases often had direct contact with wildlife prior to becoming symptomatic for EVD. The index case dates were determined by the date of symptom onset for the index patient. If this date was unavailable, the date of death of the index patient was used. In four cases only the month of an index case could be determined.

Additional index cases were identified by considering separate epidemic chains of transmission. Within the EVD outbreaks in the Republic of the Congo and Gabon are multiple spillover events, characterized by separate virus strains and epidemic chains of transmission [40, 41]. Index case patient demographics were determined from the literature. Research studies and case reports were examined to link index patients to potential sources of spillover, all of which were circumstantial.

The majority of the coordinates that we determined for spillover event locations corresponded to the index point locations in a recently created EVD database, which contains details about some spillover events [39]; however we identified additional spillover events that were not described in the database. We included the locations of index cases in Meliandou (Guinea) and Boende (DRC) that were not included in the database. We also used adjusted locations for the villages of Mwembe and Nakisamata (S1 Text).

### Environmental Data

Climate and terrain data were used to construct the ENMs. Layers of rasterized climate data of 19 bioclimatic variables as well as elevation came from the WorldClim database [42], which averages values from 1950–2000 at a spatial resolution of 30 arc seconds. We point sampled the elevation as well as long-term monthly mean rainfall and temperature at spillover locations from the WorldClim dataset in QGIS [43].

To look for seasonal relationships, we gathered the monthly mean precipitation during the month and year at the location where a spillover event occurred. We used the GPCP Version 2.2 Combined Precipitation Data Set provided by the NOAA/OAR/ESRL PSD, Boulder, Colorado, USA (available at http://www.esrl.noaa.gov/psd/). The GPCP data set has a spatial coverage of 2.5° latitude X 2.5° longitude, and uses a combination of satellite and gauge data to calculate mm precipitation per day [44]. Monthly values in the dataset are from 1979-October 2014. Therefore, we could not obtain precise monthly precipitation data for the 3 spillover events prior to 1979, so we did not include these events in analyses of within season rainfall, and we used the long-term monthly mean precipitation for classifying them into wet or dry seasons.

Temperature data for the specific month of a spillover event was gathered from the GHCN CAMS Gridded 2m Temperature (Land) dataset also provided by the NOAA/OAR/ESRL PSD, which has a resolution of .5° latitude X .5° longitude and contains monthly mean land surface temperatures from 1948 to October 2012 [45].

Vegetation and land cover were determined by mapping spillover event locations on raster maps from the Global Landcover facility (available at http://glcfapp.glcf.umd.edu). We used a global map with a spatial resolution of 225 seconds and fourteen land cover classes developed from NOAA-AVHRR satellite images from 1981–1994. We point sampled each location in QGIS to determine the land cover classification at that geographic location.

### Ecological Niche Modeling

The ENMs were built using Maximum Entropy Species Distribution Modeling (MaxEnt), version 3.3.3k [46]. The MaxEnt program applies a machine learning method to estimate the distribution of a species under maximum entropy in geographic space using environmental factors as covariates and presence-only data as inputs [47]. We chose MaxEnt over other ENMs because it is robust with small numbers of occurrences and presence-only data [48].

Models were built to determine suitable habitat for ebolaviruses using spillover events as presence-only inputs. We used the 20 environmental covariates clipped to mainland Africa for our models and analyses because TAFV, BDBV, SUDV and EBOV spillovers have only occurred within mainland Africa. We also created models with a global environmental extent for comparison. Because our aim was to characterize the environments where different ebolavirus spillover events have occurred, we did not make assumptions about sampling or the density of the population. Instead of designing the models to provide probabilistic output, we used our models as indices of habitat suitability [13].

MaxEnt can use a subset of presence points to train the model, while reserving a subset to test the predictive strength of the model. Iteratively leaving out a single occurrence point, training the model, and then testing whether that point is included in the model, works well for determining the predictive ability of a model with a small sample size [49]. Therefore, we used a leave-one-out cross-validation method for each species, in which for a sample size n of spillover locations for each species, we divided the data into n equal size folds and kept one fold out to test the model. We repeated this process n times and then averaged the models for each species.

In addition to these sampling changes, the MaxEnt models were run on the default parameters with the cumulative output and the jackknife approach for comparing environmental covariates. The cumulative output reflects habitat suitability, where the probability of occurrence in each cell is the sum of the probability in that cell as well as all other cells with lesser or equal probability [49]. The minimum training presence and the 10 percentile training presence were compared as thresholds to determine which regions were suitable or unsuitable for the respective species. To test whether the models were statistically significant at predicting presence locations compared to random, we created a program for the statistical test described by Pearson et al. 2006 [49] (available at: https://github.com/AndrewJudson/jackknife). We calculated the percent overlap of the models by dividing the area of overlap by the total area of both models. Traditional niche overlap statistics such as Schoener’s *D* and *I* were not calculated because these assume a probability distribution for the species [50], whereas our models predicted habitat suitability.

Reducing the number of environmental covariates in ENMs enables researchers to determine which covariates are driving the model. We chose to use the same covariates across the different models so that we could compare the models with each other. We used a hierarchical approach and correlation matrix to remove covariates from the initial 20 that did not contribute to the models of either ebolavirus and were highly correlated with each other. We removed covariates as long as there were no changes from the original models. For analyses and mapping, we used the models with all 20 covariates.

### Statistical Analysis

All statistical tests to determine whether the spillover events of a particular ebolavirus were more associated with certain ecological conditions were done using Fisher’s exact tests. In order to compare the differences in mean elevation, temperature, or precipitation at spillover locations, two-tailed Welch’s t-tests were used. All statistical analyses were performed in R [51], and a significance level of p < 0.05 was used.

## Supporting Information

S1 Text Worldwide habitat suitability of EBOV and SUDV spillover events. Distributions of potential reservoir and secondary host species overlaid on EBOV and SUDV spillover habitats. Additional methods for locating index cases.
(PDF)
