# Supplementary material for: Ecological Contexts of Index Cases and Spillover Events of Different Ebolaviruses
Source: PLoS Pathog. 2016 Aug 5;12(8):e1005780. doi: 10.1371/journal.ppat.1005780 (PMC4975397; doi:10.1371/journal.ppat.1005780)
Supplement: S1 Text — Distributions of potential reservoir and secondary host species overlaid on EBOV and SUDV spillover habitats. Additional methods for locating index cases. (PDF) [file ppat.1005780.s001.pdf]

Supplemental Text

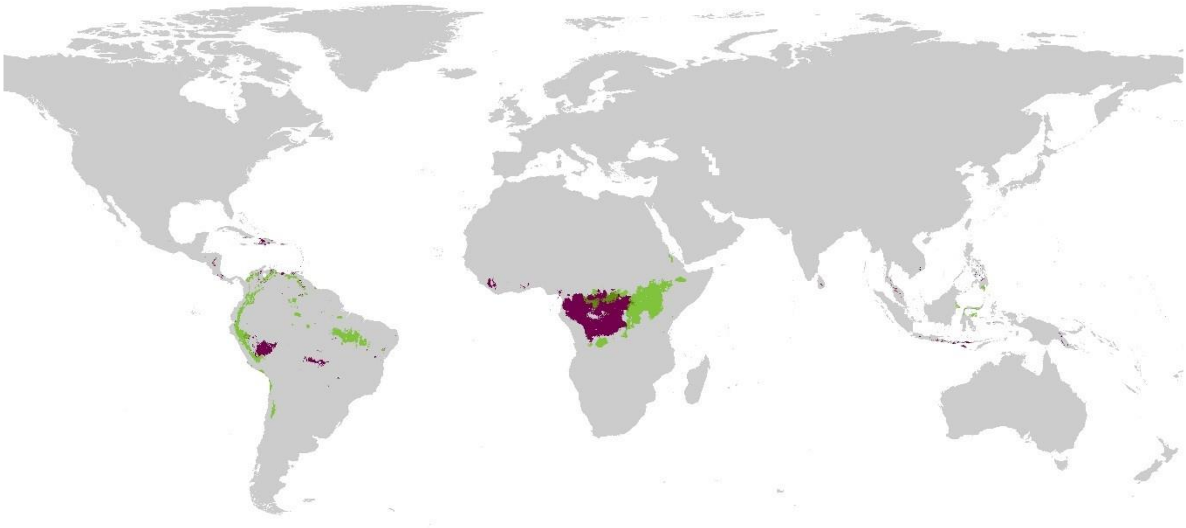

Figure A. Worldwide habitat suitability of EBOV and SUDV spillover events based on the minimum training presence of spillover events. MaxEnt models were created using the same parameters as those in Figure 2 except that global environmental covariates were used instead of those clipped to mainland Africa.

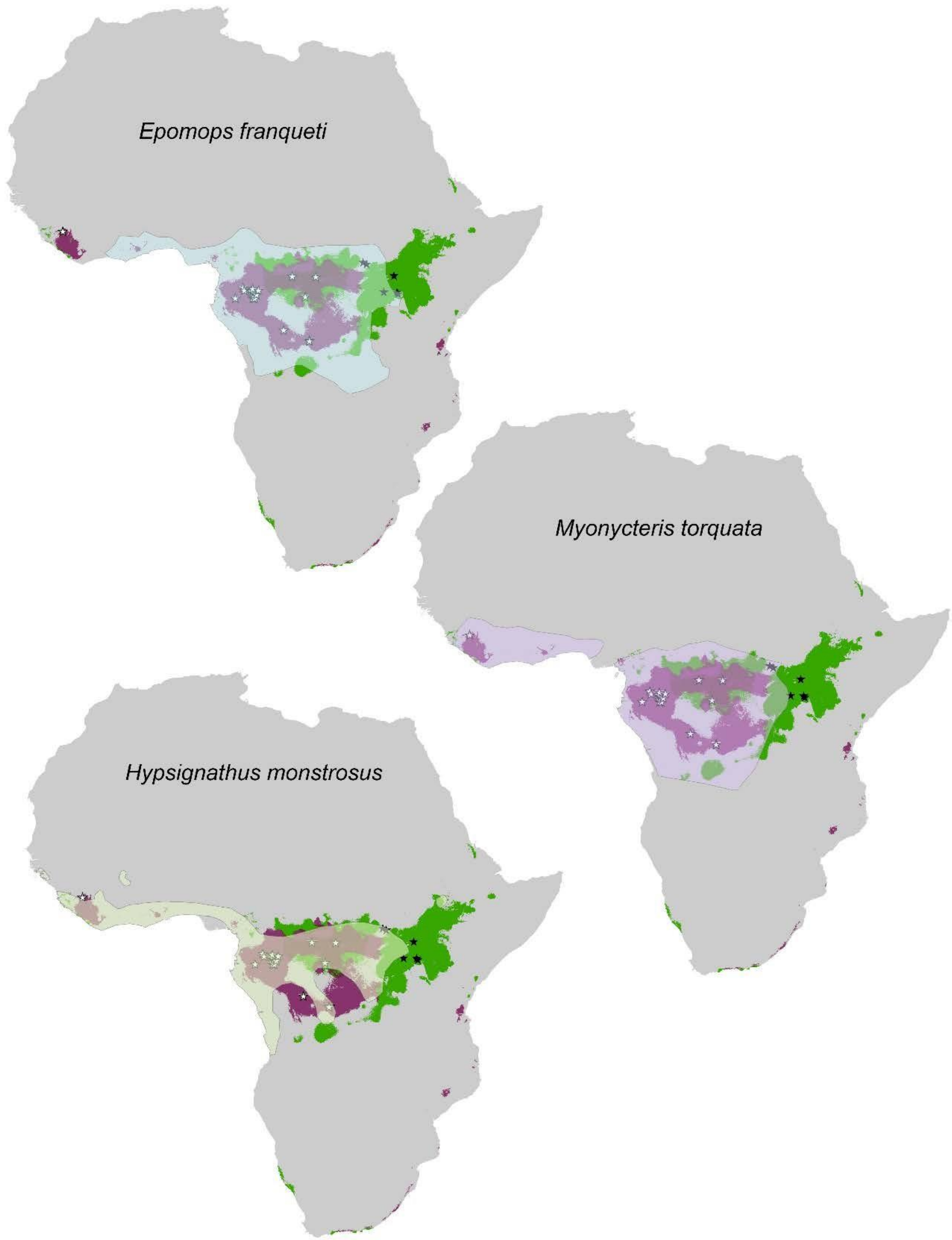

Figure B. Geographic distributions of EBOV RNA positive bat species overlaid on African EBOV and SUDV spillover habitats.

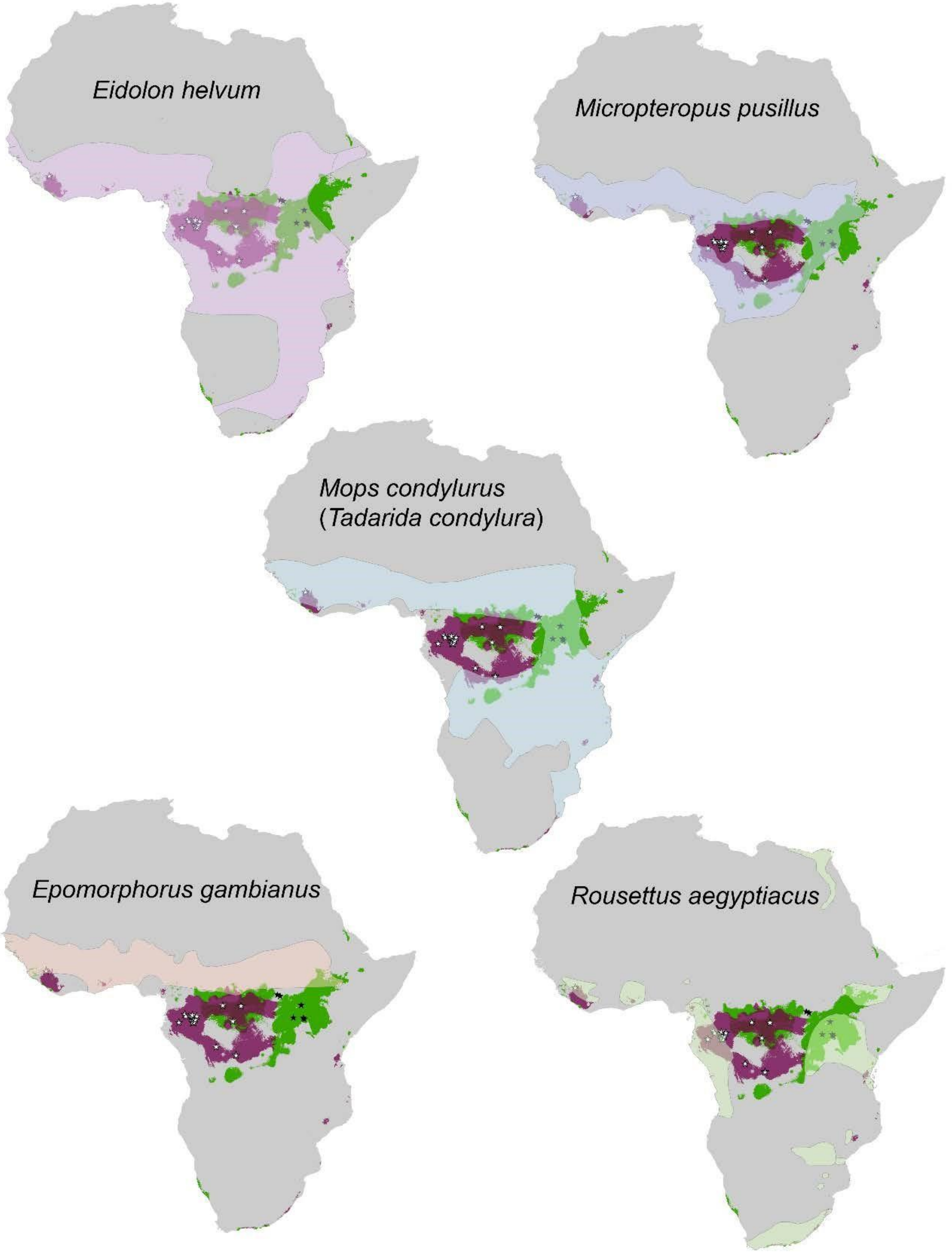

Figure C. Geographic distributions of EBOV serologically positive bat species overlaid on African EBOV and SUDV spillover habitats.

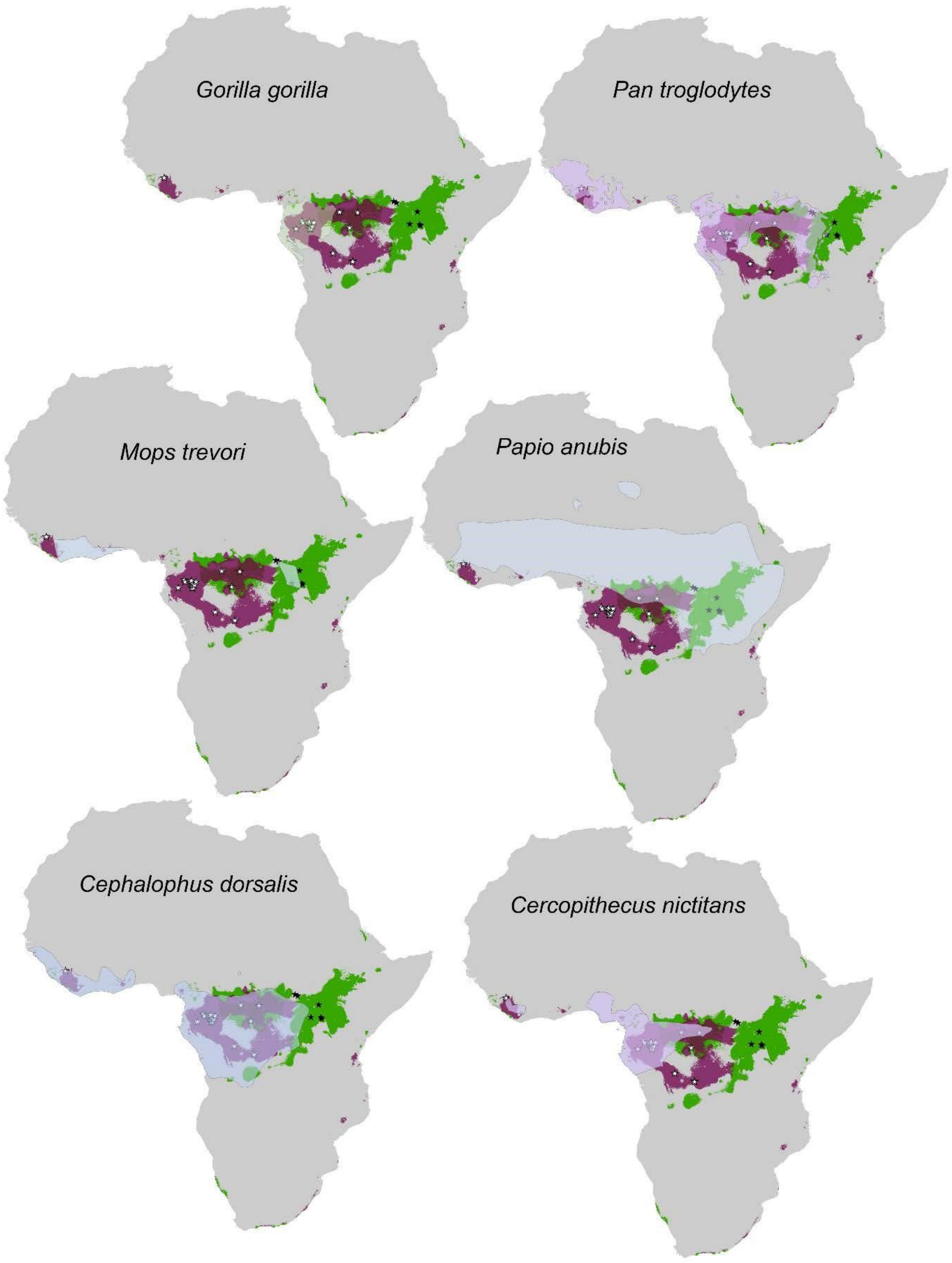

Figure D. Geographic distributions of animal species anecdotally associated with ebolavirus spillover events overlaid on African EBOV and SUDV spillover habitats.
Supplemental Experimental Procedures

Locating spillover index cases

We used adjusted locations for the villages of Mwembe and Nakisamata. The former had been estimated by the authors of the database; however, we believe that we were able to locate coordinates for Mwembe (available at http://www.geonip.com/geolocation/mwembe-bandundudemocratic-republic-of-the-congo/8447190), and the latter was another estimate by Mylne et at. 2014 that placed the Nakisamata village in a river, so we adjusted the coordinates to locate the village within the land of the Ngalonkalu parish. We also used the coordinates of Luebo and Isiro from Google Earth, which differed slightly from the center of the Luebo and Isiro polygons in the database.
